# Previous stress causes a contrasting response to cadmium toxicity in the aquatic snail *Potamopyrgus antipodarum*: lethal and behavioral endpoints

**DOI:** 10.1007/s11356-022-24932-3

**Published:** 2023-01-11

**Authors:** Álvaro Alonso

**Affiliations:** Universidad de Alcalá, Facultad de Ciencias, Departamento de Ciencias de la Vida, Unidad de Ecología, Biological Invasions Research Group, Universidad de Alcalá, Plaza de San Diego S/N, Madrid 28801 Alcalá de Henares, Spain

**Keywords:** Conductivity, Behavioral activity, Cadmium, Ecotoxicological bioassays, Aquatic toxicity, Invertebrate

## Abstract

In aquatic ecosystems, animals are often exposed to a combination of stressors, including both natural and anthropogenic factors. Combined stressors may have additive or interactive effects on animals, either magnifying or reducing the effects caused by each stressor alone. Therefore, standardized bioassays can lead to overestimations or underestimations of the risk of toxicants if natural stressors are not bear in mind. The inclusion of natural stress in laboratory bioassays may help to extrapolate the laboratory results to ecosystems. This study assesses the effects of successive exposure to two sources of stress (high water conductivity and cadmium toxicity) on the behavior and survival of the aquatic snail *Potamopyrgus antipodarum* (Tateidae, Mollusca). I conducted a bioassay consisting on exposure to high conductivity (5000 mg NaCl/L, 7 days), followed by exposure to cadmium (0.03, 0.125, and 0.25 mg Cd/L for 7 days) and by a post-exposure period (7 days). Mortality, inactivity, and the time to start activity of active animals were monitored in each animal. In general, cadmium lethality was higher in animals previously undergoing high conductivity than in non-stressed ones. Previously stressed animals showed longer time to start activity, with a noticeable effect at the two highest cadmium concentrations. Animals submitted to the two highest cadmium concentration both, stressed and non-stressed, showed a moderate recovery during the post-exposure period. It is concluded that previous stress caused a worsening of the cadmium toxicity on the aquatic snail *Potamopyrgus antipodarum*, which is especially noticeable for mortality. However, there was no interactive effect between cadmium and conductivity on snail activity, which may be indicative of recovery after cadmium exposure regardless the previous stress suffered by the snails.

**Supplementary Information:**

The online version contains supplementary material available at 10.1007/s11356-022-24932-3.

## Introduction

In natural aquatic ecosystems, organisms are usually exposed to sub-optimal conditions (Heugens et al. 2001; Holmstrup et al. 2010). In fact, extreme events such as desiccation, oxygen depletion, low food availability, or strong temperature oscillations are frequent (Bryant et al. 1985; Heugens et al. 2001; Ferreira et al. 2008; Holmstrup et al. 2010; Laskowski et al. 2010; Curtis and McGaw 2012; Freitas et al. 2016; Paudel et al. 2018; Russo et al. 2018; Cereghino et al. 2020; Radovanović et al. 2021). Physiological and behavioral changes help animals to face these stresses (Heugens et al. 2001; Holmstrup et al. 2010; Silva et al. 2018; Araujo et al. 2019). However, some human activities are increasing the concentrations of natural compounds (nutrients, some metals, etc.) and xenobiotics in the environment, which may affect animal ability to adjust to natural stresses (Russo et al. 2018; Meng et al. 2020). Natural and anthropogenic toxicant stressors acting simultaneously or in a sequential exposure may have synergic, additive, or antagonist effects on animals (Holmstrup et al. 2010; Laskowski et al. 2010; Russo et al. 2018; Gomez-Isaza et al. 2020; Meng et al. 2020; Hawkey et al. 2021). In fact, natural stressor can modify the bioavailability of the toxicant, the toxicokinetics, and the physiological capacity of organisms to face the toxicants (Holmstrup et al. 2010; Laskowski et al. 2010). Therefore, ecotoxicological risk assessments based on animal response to isolated stressors can overestimate or underestimate the risk of toxicants if the natural stressors are not borne in mind (Russo et al. 2018; Meng et al. 2020).

In laboratory bioassays, animals are usually bred and exposed to toxicants under optimal environmental conditions (such as temperature, light, and food) (Holmstrup et al. 2010; Laskowski et al. 2010; Alonso 2021). This is a requisite to understand the mode of action and the effects of toxicants on the organisms (Holmstrup et al. 2010). However, to extrapolate the results to natural ecosystems, interactions between environmental stressors and toxicants need to be assessed (Laskowski et al. 2010; Russo et al. 2018). Heugens et al. (2001) reviewed how several factors (temperature, salinity, and nutritional state) modified the sensitivity of animals to toxicants and found that, when the environmental conditions were close to the tolerance limits, animals were more vulnerable to chemical stress. Holmstrup et al. (2010) reviewed the type of interaction between environmental stress (heat, cold, desiccation, etc.) and toxicants and found synergic effects (i.e. greater effect than expected from the sum of isolated effects) in over 50% of the case studies. Russo et al. (2018) showed that bioassays consisting on sequential exposure to toxicants and environmental stressors should consider for a realistic assessment of toxicant effects. To assess potential interactions between different types of stressors (including toxicants), bioassays should use different species and endpoints, from sub-organism variables (enzymatic activities, antioxidative process, etc.) to individual and population parameters (behavior, growth, mortality, etc.) (Das and Khangarot 2010; Lushchak 2011; Silva et al. 2018; Araujo et al. 2019). Among these parameters, mortality has been widely used in ecotoxicological bioassays, because it allows the comparison of sensitivities between species, it is conducted at short-term with low cost, and it is frequently used in environmental risk assessment (Scott and Sloman 2004; Alonso and Valle-Torres 2018). However, other parameters present contrasting advantages. For instance, behavioral parameters are usually conducted at lower toxicant concentrations, and they present higher sensitivity than mortality bioassays (Melvin and Wilson 2013; Alonso 2021). Additionally, behavioral responses are the main animal mechanisms to face changes in the environment, including biotic and abiotic factors (Hellou 2011; Melvin and Wilson 2013; Alonso 2021). Thus, if we are assessing whether or not there is an interaction between environmental stress and toxicants, behavioral variables should be considered. However, this type of laboratory bioassays are relatively scarce.

Among aquatic organisms, mollusks represent a good indicator of environmental health because they are generally sensitive to a wide range of toxicants, including both inorganic to organic ones (Duft et al. 2003; Oehlman et al. 2007; Das and Khangarot 2010; Alonso and Valle-Torres 2018). Besides, several behavioral endpoints have been monitored in an ample range of mollusk species. For instance, feeding and movement variables have been amply used to assess the sensitivity of gastropods to different toxicants and other sources of stress (Snider and Gilliam 2008; Hedgespeth et al. 2018; Alonso and Valle-Torres 2018; Alonso 2021). From an ecological perspective, movement behaviors, such as time to reach the food, swimming capacity, and time to start normal activity, are directly related to animal fitness (Scott and Sloman 2004; Snider and Gilliam 2008; Alonso and Camargo 2009a; Hellou 2011; Silva et al. 2018; Alonso 2021). Therefore, the use of movement as endpoint may contribute to a more realistic assessment of the combined effects of several stressors.

Cadmium is a toxicant amply used in ecotoxicology (Wright and Welbourn 1994; Ferreira et al. 2008; Alonso et al. 2010; Das and Khangarot 2010; Alonso and Valle-Torres 2018). It is a non-essential metal for organisms often causing toxicity at low concentrations (Wright and Welbourn 1994). In the present study, cadmium has been chosen as a model toxicant because of its extreme toxicity and widespread use. Additionally, in the last decades, there has been a worrisome increase in the concentrations of ions in freshwater ecosystems associated to salinization (Bryant et al. 1985; Cañedo-Argüelles et al. 2012; Cañedo-Argüelles 2020), being another source of stress that freshwater animals must face. Given the abundant information on both sources of stress (cadmium and salinity) they may be suitable for testing their sequential effects on animal behavior and their interactions under laboratory conditions.

The aim of the present study was to assess the sequential effects of two sources of stress (salinity, by means of a high conductivity, and metal contamination with cadmium) on the behavior and survival of an aquatic snail *Potamopyrgus antipodarum* (Tateidae, Mollusca). A bioassay consisting on a exposure to high conductivity, followed by exposure to cadmium and by a recovery period, was conducted. Animals previously exposed to a high conductivity were expected to be more sensitive to cadmium and to have a worse post-exposure recovery than animals not exposed to high conductivity. Additionally, mortality and behavioral endpoints were compared to elucidate if they presented a similar trend under a sequential exposure to stress.

## Materials and methods

### Reagents and water culture

Cadmium solutions were prepared from a stock solution of CdCl_2_ (1000 mg Cd/L) (CdCl_2_, SIGMA ALDRICH 655198-5G MKBB2360, purity of 99.99% Steinheim, Germany). Standardized USEPA water (96 mg NaHCO_3_, 60 mg CaSO_4_·2H_2_O, 4 mg KCl, 122.2 mg MgSO_4_, per liter of deionized water plus 10 mg CaCO_3_ per liter) was used for the culture (USEPA 2002).

### Animal culture

Animals used for the bioassay were obtained from a culture started at the University of Alcalá (Laboratory of Ecology, Department of Life Sciences). The culture was started in 2009 with animals collected in the upper reach of the Henares River (Guadalajara, Spain). Animals were kept in 60 L aquaria with 0.10 mg of dry food per animal and day (50% fish food Tetra- Menü© GmbH, Melle, Germany + 50% Sera © Spirulina Tabs GmbH, Heinsberg, Germany). The 10% of water culture was renewed every 2 weeks to ensure a good water quality. Water was aerated using aquarium filters (filtered water was mixed with air through a small waterfall).

### Experimental design

#### High-conductivity bioassay

A first bioassay was conducted to cause stress on animals by means of a high conductivity (see supplementary material S1). Four hundred adult animals (3.9 ± 0.2 mm in shell length) were collected from the culture. Snails were placed randomly in six aquaria (1 L) (65–67 animals per aquarium). Three aquaria were filled with 1 L standardized USEPA water and the remaining aquaria with a sodium chloride solution in standardized USEPA water (5000 mg NaCl/L, ALDRICH, Denmark, no. SZBE2110V, > 99.8% purity). The sodium chloride concentration was selected after a previous test to determine a non-lethal concentration that caused a decay in snail behavior. Two treatments were conducted: non-stressed animals (in USEPA water) and stressed animals (with high conductivity by means of sodium chloride solution). Animals were exposed to these treatments during 7 days at 18 °C (climatic chamber ANSONIC) (12 h:12 h photoperiod). After 3 days of exposure, animals were fed with food pellets (JBL, GmbH&Co., Germany) (approx. 0.5–0.6 g per aquarium for 3 h), and subsequently, water and sodium chloride solution from both treatments were renewed.

#### Cadmium bioassay

All animals survived to the first bioassay. Immediately after, a random selection of previously stressed and non-stressed animals were exposed to cadmium for 7 days in a second bioassay (see supplementary material S1). Four treatments were set up: control, 0.03, 0.125, and 0.25 mg Cd/L. Cadmium concentrations were selected, based on a previous study with the same species (Alonso and Valle-Torres 2018). Each treatment was replicated 8 times. In each replicate (a glass vessel with 100 mL of solution), 6 animals were used. The toxicant solutions and control water were renewed after 3 days. Before the water renovation, animals were fed with food pellets (JBL, GmbH&Co., Germany) (approx. 0.1 gr per replicate for 3 h). After 7 days of exposure to cadmium, surviving animals were transferred to standardized USEPA water (USEPA 2002) for 7 additional days of post-exposure. Therefore, the second bioassay lasted 14 days at 18 °C in climatic chamber (ANSONIC). After 10 days, water was renewed again, and animals were fed in the same way. Actual cadmium concentrations were monitored at days 0, 3 (before and after water renovation), and 7 of exposure period by means of a spectrophotometer (Spectroquant© NOVA60, Merck, KGaA, 64293 Darmstadt, Germany) and the Spectroquant Cadmium Test (1.01745.0001, Spectroquant©, Merck, KGaA, 64271 Darmstadt, Germany). This analytical method is based on the reaction of cadmium ions with 1-(4-nitrophenyl)-3-(4-phenylazophenyl)triazene which form a red complex. The intensity of the red complex is spectrophotometrically quantified. The sensitivity of this method ranged between 0.002 and 0.5 mg Cd/L. The analytical quality assurance was checked following the recommendations of Spectroquant Cadmium Test (1.01745.0001, Spectroquant, Merck©).

#### Monitoring variables

During the second bioassay, mortality, inactivity, and time to start activity were assessed at days 0 (before exposure to cadmium), 3, and 7 of exposure to cadmium and at days 10 and 14 (i.e. days 3 and 7 of post-exposure to cadmium) (see supplementary material S1). The time to start activity (in seconds) was monitored with a chronometer as the time spent by each animal to start the sliding movement (Alonso 2021). In the vessel of each replicate, each animal was taken up with forceps and placed in the center of the vessel with the operculum downwards (Alonso 2021). If the animal did not move after 120 s, it was considered as inactive. Therefore, percentage of inactive animals and the time to start activity were two behavioral variables. If no reaction was observed when the operculum was touched with forceps, the inactive animal was considered as dead (lethal variable) (Alonso 2021). All variables were monitored using a stereomicroscope (MOTIC® SMZ-168) equipped with optic fiber beam (Jenalux® 150). The water temperature (°C) and dissolved oxygen (mg O_2_/L) were measured at 0, 3, 7, and 14 days of the second bioassay (0, 3, and 7 in the first bioassay) by means of an oximeter (Crison® Oxi 45+). With the same periodicity, water conductivity (microS/cm) was measured with a conductivimeter (Crison® CM35+) and pH with a pHmeter (Crison micropH 2001, ALELLA 08328). After finishing the second bioassay, the shell length of every snail was measured using a micrometer installed in a stereoscopic microscope (MOTIC® SMZ-168).

### Statistical analysis

To assess the effects of stress, cadmium, time, and their interactions on the dependent variables (percentage of mortality, percentage of inactive animals, and the time to start activity of *P. antipodarum* in the active animals), a three-way repeated-measure ANOVA was conducted. Time (0, 3, 7, 10, and 14 days) was used as intrasubject factor; the cadmium treatments (control, 0.03, 0.125, and 0.25 mg Cd/L) and stress treatment (stressed and non-stressed) were intersubject factor. Time to start the activity was only considered for animals with less than 120 s of activity. After the three-way repeated-measure ANOVA, cadmium treatments were compared with the control with a pairwise *t*-test with Bonferroni correction. All statistical analyses were conducted using R 3.5.1. Software (R Core Team 2019).

## Results

In the first bioassay, the conductivity (mean ± SD) (*n* = 9) was 9.51 ± 0.06 miliS/cm and 351.8 ± 8.2 microS/cm in the stressed and non-stressed groups, respectively. Water temperature (*n* = 9) was 17.6 ± 0.4 °C for non-stressed group and 17.5 ± 0.5 °C for stressed group, mean (*n* = 9) dissolved oxygen 9.29 ± 0.1 mg O_2_/L for non-stressed group and 9.27 ± 0.1 mg O_2_/L for stressed group, and mean (*n* = 9) pH 8.01 ± 0.04 for non-stressed group and 8.02 ± 0.07 for stressed group. The physical-chemical parameters of the cadmium bioassay (mean ± SD) of non-stressed animals for each treatment (control and increasing cadmium concentrations) (*n* = 3–4) were 17.5 ± 0.3, 17.5 ± 0.6, 17.7 ± 0.4, and 17.6 ± 0.8 °C of water temperature; 9.3 ± 0.1, 9.3 ± 0.0, 9.4 ± 0.1, and 9.6 ± 0.1 mg O_2_/L of dissolved oxygen; 356 ± 10.3, 346.8 ± 9.3, 350.7 ± 12.7, and 345.7 ± 12 microS/cm of conductivity; and 8.1 ± 0, 8.0 ± 0.0, 8.0 ± 0.1, and 8.0 ± 0.0 of pH. The physical-chemical parameters of the cadmium bioassay (mean ± SD) of stressed animals for each treatment (control and increasing cadmium concentrations) (*n* = 3–4) were 17.6 ± 0.5, 17.5 ± 0.1, 17.6 ± 0.2, and 17.6 ± 0.2 °C of water temperature; 9.5 ± 0.2, 9.3 ± 0.1, 9.3 ± 0.1, and 9.3 ± 0.0 mg O_2_/L of dissolved oxygen; 345.7 ± 10, 353.0 ± 2.6, 352.7 ± 6.8, and 350.0 ± 9 microS/cm of conductivity; and 8.1 ± 0.1, 8.1 ± 0.0, 8.1 ± 0.0, and 8.1 ± 0.0 of pH. The mean (± SD) (*n* = 9–12) actual concentrations of cadmium in the non-stressed groups were 0.028 ± 0.008, 0.108 ± 0.014, and 0.238 ± 0.019 mg Cd/L and in the stressed groups were 0.029 ± 0.002, 0.112 ± 0.006, and 0.215 ± 0.018 mg Cd/L. These concentrations were very similar to the nominal concentrations (0.03, 0.125, and 0.25 mg Cd/L) so the latter were used in the results. Cadmium concentrations in the controls was less than 0.01 mg Cd/L (*n* = 21). Animals used in the bioassays presented a mean shell length (mean ± SD) (*n* = 128) of 3.9 ± 0.2 mm.

**Fig. 1:**
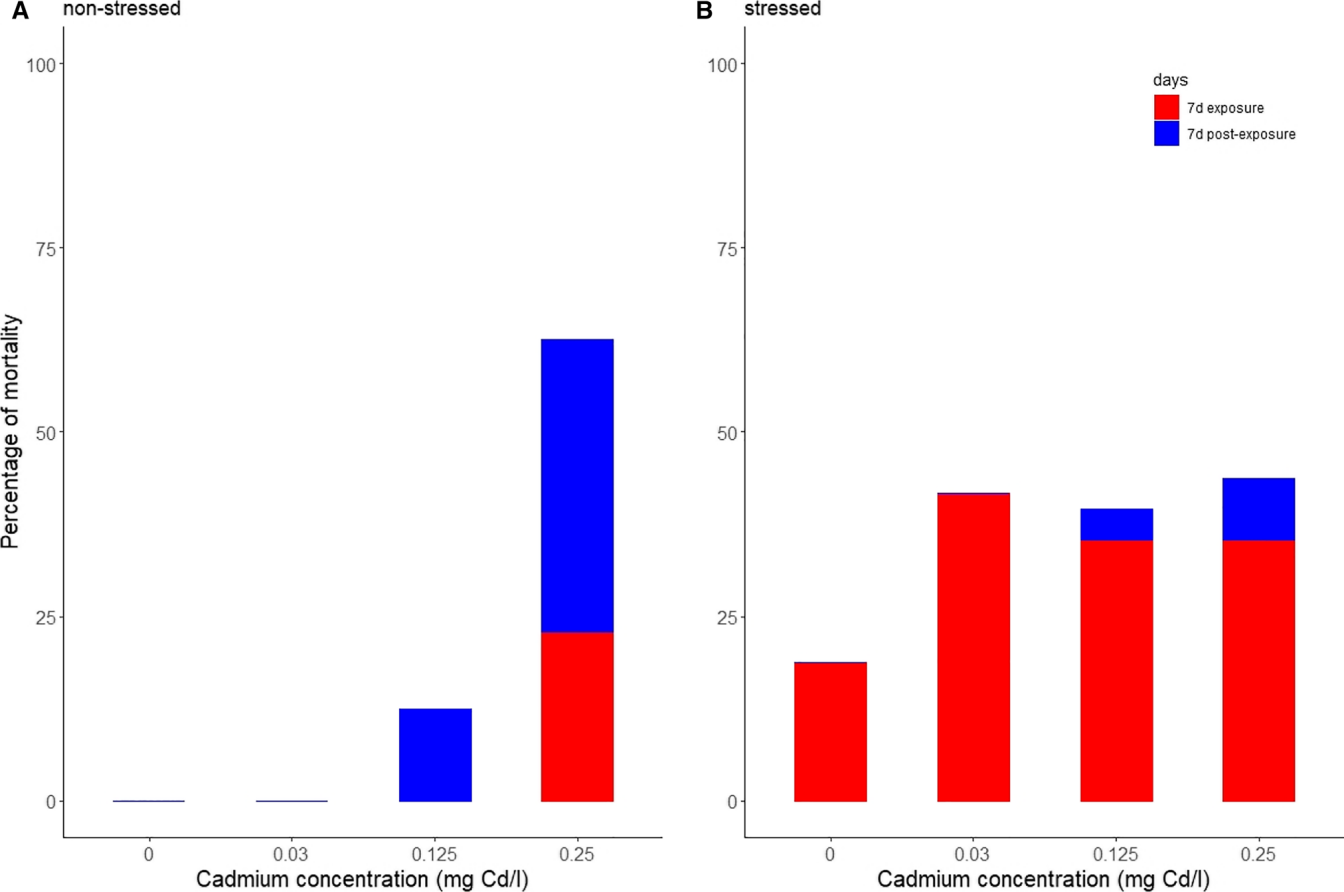
Percentage of cumulative mortality of *Potamopyrgus antipodarum* for each stress treatment (non-stressed **A**; stressed **B**), for each cadmium concentration (0, 0.03, 0.125, and 0.25 mg Cd/L) and after 7 days of exposure to cadmium (red columns) and 7 days of post-exposure to cadmium (blue columns). The mortality in all cadmium concentrations was significantly different from control (*p* < 0.05; pairwise test with Bonferroni correction). See Table 1 for results of the statistical analysis

No animals died in the control group of non-stressed animals during the cadmium bioassay (Fig. 1). After 7 d of cadmium exposure, stressed animals of the control showed a mortality below 25% (Fig. 1). This result showed that the treatment of high conductivity was effective to cause certain mortality. The lowest cadmium concentration did not cause mortality to non-stressed animals (Fig. 1). However, all cadmium concentrations caused a similar mortality to stressed animals (Fig. 1), only being slightly higher in the two highest concentrations after 7 d of post-exposure (Fig. 1). For mortality, all treatments and their interactions were statistically significant (*p* < 0.05; repeated-measures three-way ANOVA, Table 1). In general, the effect of cadmium was higher in stressed than in non-stressed animals during the exposure period, the opposite being true during the post-exposure period (Fig. 1).

**Table 1:** Results for the repeated-measure three-way ANOVA where the stress treatment (=stress) (stressed, non-stressed) and cadmium concentration (=Cd) (control, 0.03, 0.125, and 0.25 mg Cd/L) were the intersubject factors; time (=Time) (0, 3, 7, 10, and 14 days) was the intrasubject factor; and cumulative mortality, percentage of inactive animals, and time to start movement were the dependent variables

Source of variation	*df*^a^	*F*	*p*
Mortality			
Intrasubject factor			
Time	2.1	81.1	< 0.001
Time × Cd	6.3	9.93	< 0.001
Time × stress	2.1	19.4	< 0.001
Time × Cd × stress	6.3	7.46	< 0.001
Intersubject factors			
Cd	3	12.8	< 0.001
Stress	1	55.4	< 0.001
Cd × stress	3	6.28	< 0.001
Inactive animals			
Intrasubject factor			
Time	2.7	12.4	< 0.001
Time × Cd	8.2	8.81	< 0.001
Time × stress	2.7	6.39	< 0.001
Time × Cd × stress	8.2	8.28	< 0.001
Intersubject factors			
Cd	3	21.5	< 0.001
Stress	1	8.65	< 0.001
Cd × stress	3	14.5	< 0.001
Time to start activity			
Intrasubject factor			
Time	3.3	8.54	< 0.001
Time × Cd	9.9	4.7	< 0.001
Time × stress	3.3	29.9	< 0.001
Time × Cd × stress	9.9	3.05	< 0.001
Intersubject factors			
Cd	3	31.2	< 0.001
Stress	1	76.7	< 0.001
Cd × stress	3	1.15	0.339

*df*, degrees of freedom

^a^*df* were corrected for sphericity using the Grennhouse-Geisser approach

**Fig. 2:**
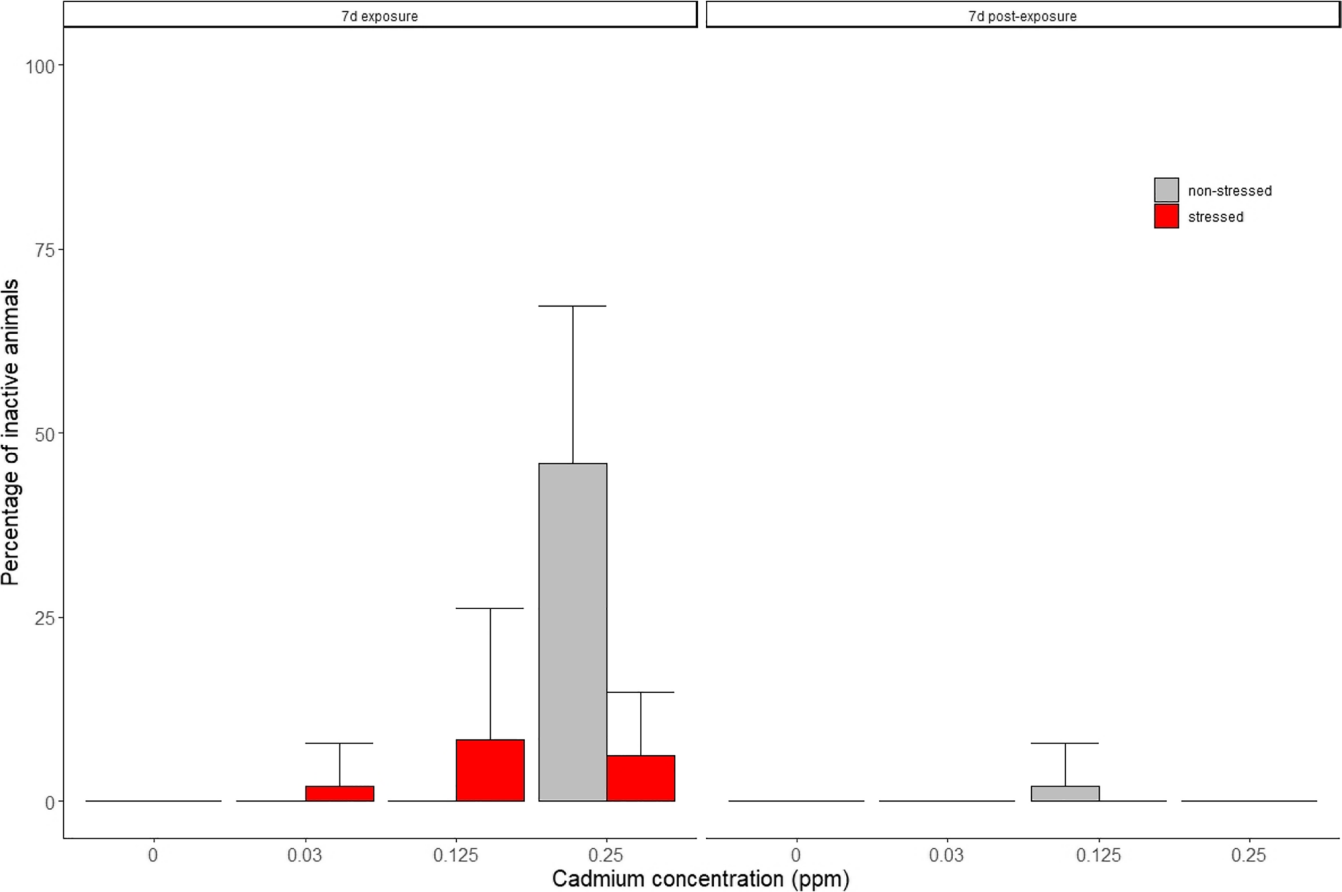
Percentage of inactive animals for the exposure period (7 days, left panel) and post-exposure period (7 days, right panel), stress treatment (non-stressed in gray bars and stressed in red bars), and for each cadmium concentration (0, 0.03, 0.125, and 0.25 mg Cd/L). The percentage of inactive animals was significantly different from control in the highest cadmium concentration (*p* < 0.05; pairwise test with Bonferroni correction). See Table 1 for results of the statistical analysis

The percentage of inactive animals was higher during the exposure period than in the post-exposure period (*p* < 0.05; repeated-measures three-way ANOVA, Fig. 2, Table 1), being negligible both among stressed and non-stressed animals in the post-exposure period. The highest cadmium concentration increased the proportion of inactive animals in the non-stressed groups (*p* < 0.05; pairwise test with Bonferroni correction, Fig. 2).

**Fig. 3:**
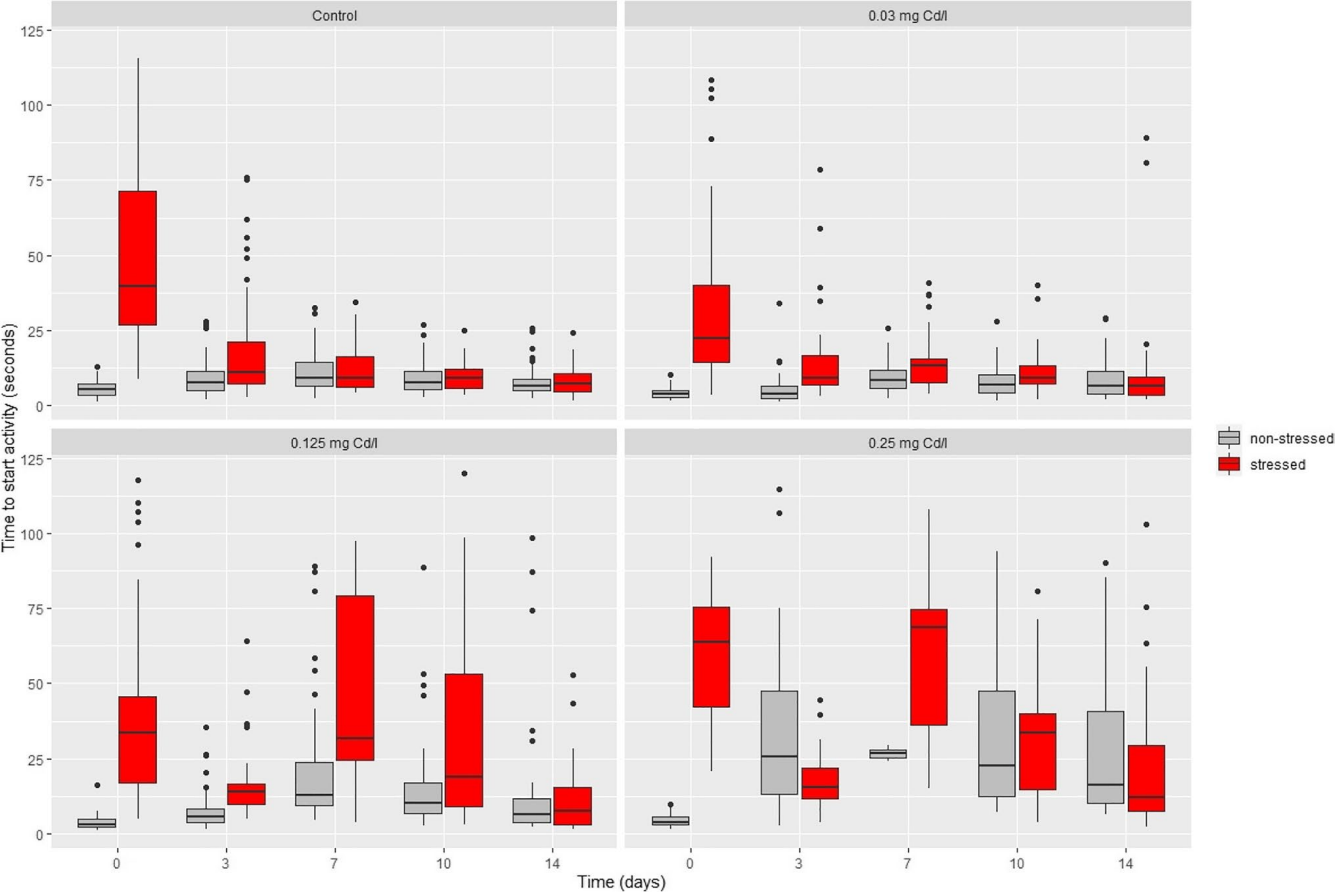
Time to start activity (in seconds) of animals in each cadmium concentration (in mg Cd/L) (control: left top panel, 0.03: right top panel, 0.125: left bottom panel, 0.25: right bottom panel), stress treatment (non-stressed in gray bars and stressed in red bars) and for each time (*X*-axis in days). The time to start movement was significantly different from the control in the highest cadmium concentration (*p* < 0.05; pairwise test with Bonferroni correction). See Table 1 for results of the repeated-measure three-way ANOVA

Time to start activity was affected by all factors (time, cadmium, and stress treatment) (*p* < 0.05; repeated-measures three-way ANOVA, Fig. 3, Table 1). The highest cadmium concentration caused an increase in this variable (*p* < 0.05; pairwise test with Bonferroni correction, Fig. 3). In general, stressed animals took a longer time to start activity than non-stressed animals, with a noticeable effect of the two highest cadmium concentrations (Fig. 3). Control stressed animals showed a longer time to start activity than control non-stressed animals (Fig. 3). In general, animals of the two highest cadmium concentrations showed a moderate recovery during the post-exposure period (10 and 14 days of the second bioassay), both among stressed and non-stressed animals (Fig. 3).

All interactions were statistically significant (*p* < 0.05; repeated-measures three-way ANOVA, Table 1) excepting the cadmium × stress treatments for the time to start activity (*p* = 0.339; repeated-measures three-way ANOVA, Table 1). This is shown in Fig. 3, where the time to start activity showed the same trend among stressed and non-stressed animals (with higher activity in stressed animals) and among cadmium treatments.

## Discussion

When animals are subjected to stressed conditions, several mechanisms, including physiological, biochemical, and behavioral changes occur to maintain homeostasis (Dao et al. 2013; Bertrand et al. 2017; Fan et al. 2019; Meng et al. 2020). In natural ecosystems, animals may face different sources of both biotic and abiotic stresses, such as competition, predation risk, starvation, salinity changes, different toxicants, or thermal variation (Heugens et al. 2001; Relyea and Mills 2001; Alonso et al. 2010; Holmstrup et al. 2010; Liess and Foit 2010; Dao et al. 2013; Bertrand et al. 2017; Russo et al. 2018; Fan et al. 2019; Meng et al. 2020; Alonso 2021; Hawkey et al. 2021). These stresses may cause a depletion of energy reserves, as animals must deal with oxidative stress or generate heat shock proteins, among others (Downs et al. 2001; Alonso et al. 2010; Holmstrup et al. 2010; Dao et al. 2013; Bertrand et al. 2017; Meng et al. 2020; Cheng et al. 2021). This depletion may impair overall performance of animals, including growth, reproduction, and behavior (Calow 1991; Dao et al. 2013; Bertrand et al. 2017; Russo et al. 2018). Therefore, if during or after the exposure to a source of stress animals are submitted to an extra stress, such as a toxicant, the energy available for detoxification could be reduced, lowering its effectiveness and exacerbating the adverse effects of toxicants (Downs et al. 2001; Holmstrup et al. 2010; Russo et al. 2018). Moreover, empirical results indicate that more than 50% of the studies on the interaction between natural stressors with environmental chemicals resulted in synergistic interactions (Holmstrup et al. 2010). Indeed, several studies reported that the sequential exposure to several sources of stress caused an increase of adverse effects on several species of aquatic animals (Russo et al. 2018; Meng et al. 2020). However, antagonistic effects have also been described (Meng et al. 2020).

The response of animals exposed to natural stress and toxicants was found to vary with the species, the natural stress, and the toxicant (Meador 1993; McGee et al. 1998; Relyea and Mills 2001; Alonso et al. 2010; Ferreira et al. 2010; Holmstrup et al. 2010; Laskowski et al. 2010; Meng et al. 2020). Starved and fed individuals of the estuarine amphipod *Leptocheirus plumosus* showed a similar sensitivity to cadmium (McGee et al. 1998). On the contrary, non-starved amphipods (*Gammarus pulex*) showed a higher sensitivity to pulses of cadmium than starved animals (Alonso et al. 2010). Meador (1993) found that a decrease of whole-body lipid content may be an indicator of declining animal health, which determines the tolerance to cadmium of amphipods. Defo et al. (2019) suggested that dietary cadmium may intensify the stress caused by high water temperature on trout transcriptomic responses. In the present study, animals exposed to high conductivity showed higher mortality in low cadmium treatments than that of non-stressed animals. On the contrary, animals exposed to high conductivity showed less mortality at the end of the post-exposure period to the highest cadmium concentration than non-stressed animals. However, behavior activity did not show that interactive response. Exposure to high conductivity/salinity or variations in salinity represents an important source of stress for animals (Carregosa et al. 2014; Bertrand et al. 2017). Carregosa et al. (2014) showed that hyper salinity induces oxidative stress in bivalves, and the osmoregulation processes may suppose a rapid mobilization of energy reserves. In fact, declines of energy reserves and behavioral impartments are indicators of salinity stress in bivalves (Bertrand et al. 2017). The exposure of *P. antipodarum* to osmotic stress caused a decline of metabolic rates, enzymatic activities, and a reduction in the percentage of active snails; finally, at high salinities, this species undergoes a suppression of aerobic metabolism (Paolucci and Thuesen 2020). In the present study, in the absence of cadmium (control groups), individuals of *P. antipodarum* exposed to high salinity needed longer time to start activity than control non-stressed animals. However, stressed animals tended to recover its activity with time in the lowest cadmium treatments (and less noticeable in the highest concentrations), which is indicative of a likely improvement of physiological state. Therefore, animals previously exposed to high conductivity started the exposure to cadmium with a behavioral disorder, which may be indicative of its physiological impairment. This is supported by the study by Paolucci and Thuesen (2020), who found a low metabolic rate and a decline in enzymatic activities related to aerobic and anaerobic metabolism in individuals of *P. antipodarum* exposed to a high salinity. This may help explain the high mortality found in control and cadmium-treated stressed animals during cadmium exposure. The similar mortality among cadmium treatments for stressed animals also point at the intense stress caused by the previous exposure to high conductivity. In this sense, the decline of metabolic rate may explain a subsequent reduction in the efficacy of cadmium detoxification mechanisms, such us metal sequestration and excretion (Das and Khangarot 2010). Therefore, the stress caused by exposure to high conductivity generated a decline threshold response to cadmium, so once exceeded, very similar effects were found across cadmium treatments.

In the present study, behavioral activity (time to start activity) was recorded in animals that were active (i.e. neither dead neither inactive). Consequently, they were the most tolerant to salinity and/or cadmium among the animals of the tested population. This may explain their capacity to recover the behavior during the post-exposure period, at least in the low cadmium treatments. The gills are the main route of entry of waterborne cadmium in aquatic animals (Schill et al. 2003; Marsden and Rainbow 2004; Xu et al. 2021). Therefore, during cadmium exposure, animals can rapidly uptake cadmium, especially at high concentrations. The tolerance threshold for cadmium in the most sensitive animals could have been exceeded during exposure, causing death or inactivity. The remaining active animals may present efficient detoxification mechanisms, which could help attain a rapid recovery after exposure in both stressed and non-stressed animals. A similar trend in the tolerance after the exposure to a natural stressor was found by Meng et al. (2020) in mosquito larvae, which were less sensitive to the toxicity of the pesticide chlorpyrifos after a heat spike exposure. Both results may be explained by the survival selection caused by the previous stress which probably killed the weaker organisms (Meng et al. 2020).

Post-exposure periods in laboratory bioassays allow the monitoring of the toxicant effects in a realistic way, since delayed effects are taken into account when calculating endpoints (Schill et al. 2003; Alonso and Camargo 2009b; Alonso et al. 2010; Pais-Costa et al. 2015; Xu et al. 2021). In several previous studies, periods of post-exposure to toxicants had exhibited significant effects on survival, reproduction, growth, and behavior in different species, including *P. antipodarum* (Handy 1994; Jensen and Forbes 2001; Alonso and Camargo 2009b; Alonso et al. 2010; Azevedo-Pereira et al. 2011; Pais-Costa et al. 2015). The exposure of the freshwater amphipod *Gammarus fossarum* to cadmium for 5 days, followed by a recovery period of 15 days, showed that mortality occurred mainly in the first five days of recovery (Schill et al. 2003). Zhao and Newman (2006) showed that copper caused pervasive damage to amphipods after exposure, which means that organisms may need relatively long periods to recover. Mortality after toxicant exposure was high and related to the cumulative damage caused by copper, which is a function of both concentration and duration (Zhao and Newman 2006). For cadmium, Pascoe and Shazili (1986) found in fish that longer exposures resulted in higher mortality in post-exposure periods. The feeding rate in the amphipod *Gammarus pulex* worsened after zinc exposure (Wilding and Maltby 2006). A delayed mortality was found in *P. antipodarum* after short-term exposure to cadmium (48-96h) (Jensen and Forbes 2001). Other studies have shown similar trends (Zhao and Newman 2006; Pais-Costa et al. 2015). Therefore, the inclusion of post-exposure/recovery periods after toxicant exposure is an improvement in the realism of laboratory bioassays, as it allows a better prediction of the fate of field populations (Zhao and Newman 2006; Pais-Costa et al. 2015). In fact, in our study, the mortality among non-stressed animals at the highest cadmium concentration at the end of the recovery was more than twice that found at the end of the exposure period. This may be explained by the death of the most cadmium-sensitive animals during the recovery period, an aspect that would not occur in the more sensitive stressed animals, which could die at the beginning of the exposure to cadmium.

## Conclusions

The sensitivity to cadmium of the aquatic snail *P. antipodarum* increased after a previous exposure to high conductivity, which was especially notable for the mortality endpoint. However, the activity of snails did not respond to the interaction between conductivity stress and cadmium exposure, which may be indicative of a behavioral recovery after cadmium exposure regardless the previous stress suffered by the snails. Therefore, to increase the realism of bioassays, the post-exposure effects of toxicants and the interaction with environmental stress should be taken into account.

## Supplementary Information

Below is the link to the electronic supplementary material.

Supplementary file1 (DOCX 119 KB)

## Data Availability

Data that support the findings of this study are available from the corresponding author upon reasonable request

## Code availability

Rcode is available from the corresponding author on reasonable request
