# Supplementary figures and images for: Previous stress causes a contrasting response to cadmium toxicity in the aquatic snail Potamopyrgus antipodarum: lethal and behavioral endpoints
Source: Environ Sci Pollut Res Int. 2023 Jan 11;30(14):41348–58. doi: 10.1007/s11356-022-24932-3 (PMC10067653; doi:10.1007/s11356-022-24932-3)

**
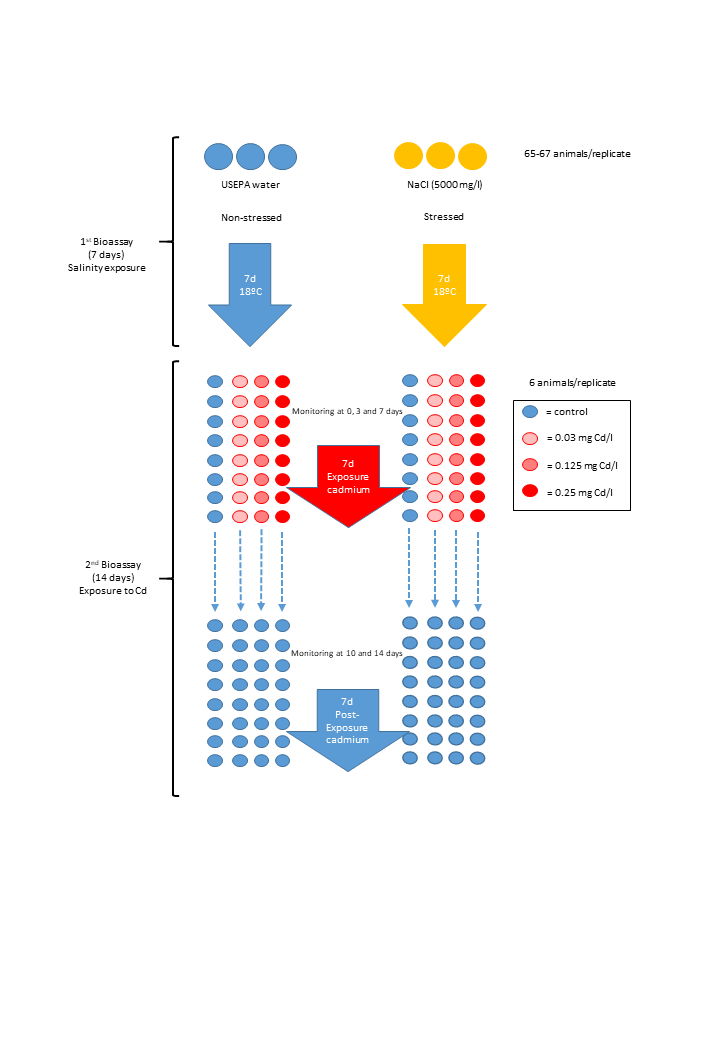
**

**Supplementary S1** Experimental design.

Supplement: Supplementary file 1 — Supplementary file1 (DOCX 119 KB) [file 11356_2022_24932_MOESM1_ESM.docx]
